# Assessment of Motor Evoked Potentials in Multiple Sclerosis

**DOI:** 10.3390/s23010497

**Published:** 2023-01-02

**Authors:** Joško Šoda, Sanda Pavelin, Igor Vujović, Maja Rogić Vidaković

**Affiliations:** 1 Signal Processing, Analysis, and Advanced Diagnostics Research and Education Laboratory (SPAADREL), Faculty of Maritime Studies, University of Split, 21000 Split, Croatia; 2 Department of Neurology, University Hospital of Split, 21000 Split, Croatia; 3 Laboratory for Human and Experimental Neurophysiology, Department of Neuroscience, School of Medicine, University of Split, 21000 Split, Croatia

**Keywords:** multiple sclerosis, TMS, line navigation, e-field navigation, navigated TMS, evoked potentials, motor evoked potentials, MEP

## Abstract

Transcranial magnetic stimulation (TMS) is a noninvasive technique mainly used for the assessment of corticospinal tract integrity and excitability of the primary motor cortices. Motor evoked potentials (MEPs) play a pivotal role in TMS studies. TMS clinical guidelines, concerning the use and interpretation of MEPs in diagnosing and monitoring corticospinal tract integrity in people with multiple sclerosis (pwMS), were established almost ten years ago and refer mainly to the use of TMS implementation; this comprises the magnetic stimulator connected to a standard EMG unit, with the positioning of the coil performed by using the external landmarks on the head. The aim of the present work was to conduct a narrative literature review on the MEP assessment and outcome measures in clinical and research settings, assessed by TMS Methodological characteristics of different TMS system implementations (TMS without navigation, line-navigated TMS and e-field-navigated TMS); these were discussed in the context of mapping the corticospinal tract integrity in MS. An MEP assessment of two case reports, by using an e-field-navigated TMS, was presented; the results of the correspondence between the e-field-navigated TMS with MRI, and the EDSS classifications were presented. Practical and technical guiding principles for the improvement of TMS studies in MEP assessment for MS are discussed, suggesting the use of e-field TMS assessment in the sense that it can improve the accuracy of corticospinal tract integrity testing by providing a more objective correspondence of the neurophysiological (e-field-navigated TMS) and clinical (Expanded Disability Status Scale—EDSS) classifications.

## 1. Introduction

Multiple sclerosis (MS) is an inflammatory autoimmune disease of the central nervous system (CNS) of an unknown cause, characterized by demyelinating white matter lesions and neuronal degeneration [1]. The prevalence of MS in the world ranges from 5 to 300 per 100,000 people, and affects women more often [2]. Relapsing–remitting form of the disease (RRMS) is the most common form. The primary progressive form of the disease (PPMS) is significantly less common and occurs in 10% of people with MS (pwMS), while the further progression of the disease indicates the transition from the relapsing–remitting form to the secondary progressive form (SPMS).

The diagnosis of MS is based on laboratory findings (e.g., cerebrospinal fluid-specific bands), oligoclonal bands, and radiologic findings (e.g., magnetic resonance imaging [MRI ≥ 1.5 T or 3T] T2 lesions of the brain and spinal cord, lesions that increase gadolinium), including the application of the 2017 McDonald criteria and the 2021 MAGNIMS-CMSC-NAIMS recommendations [3,4]. The clinical status of disability is expressed through the Expanded Disability Status Scale (EDSS) [5,6], which assesses the status of functional systems including the pyramidal–corticospinal pathway (muscle strength, limb movement), cerebellum (balance, coordination), brainstem (speech, swallowing, nystagmus), sensory pathway (sensation), visual pathway (sight), bladder and bowel function, cognitive functions (memory), and ambulation (walking measured in meters). The key functional components of the EDSS, correlating with sustained disability progression, appear to be mostly pyramidal, followed by cerebellar and sensory functional systems [7].

Various quantitative measures (i.e., the number and volume of contrast-enhancing, the volumes of T2-hyperintense and T1-hypointense lesions, and brain volume changes), derived from conventional and advanced MRI methods, have been proposed as prognostic biomarkers for MS. However, correlations between different MRI indicators and EDSS are not satisfactory, and no specific MRI measure is used as a comprehensive prognostic imaging biomarker for MS [8,9,10].

Evoked potentials (EP) represent neurophysiological measures of signal conduction in the CNS in vivo, and are used to measure the impact of MS pathology on CNS function pathways correlating with clinical status [11]. Multimodal Eps, such as somatosensory evoked potentials (SEPs), visual evoked potentials (VEPs), and motor evoked potentials (MEPs), recorded as baseline (at diagnosis), have been shown to correlate with EDSS [12]. Recent findings suggest the likely application of TMS as a subclinical MEP test that could represent a biomarker of the degree of MS disability [13,14]. Current data suggest a connection between the pathophysiological mechanisms of MS (demyelination and loss of axons) and TMS neurophysiological measures (e.g., lower amplitudes and longer latencies of MEP responses from upper and lower limb muscles, elevated resting motor threshold (RMT), and changes in specific neurophysiological measures of excitation and inhibition) [15]. Furthermore, changes in cortical excitatory and inhibitory processes in MS, assessed with TMS, appear to be evident in early disease progression, during relapse, and later during disease progression [11,15,16]. In addition, changes in neurophysiological TMS measures are associated with the clinical characteristics of MS [14,15]. It has to be noted that MEPs acquired in TMS studies in MS subjects, mainly represent the marker of the integrity of the corticospinal tract (lateral funicle of the cord known as the lateral corticospinal tract) [17] and primary motor cortices (M1). Motor mapping can also demonstrate the presence of the ipsilateral MEP corticospinal tract projections reported in congenital pathologies, including hemiplegic cerebral palsy [18,19,20,21,22] and congenital mirror movements [18,23,24,25]; this is evident in progressive immune-mediated Rasmussen encephalitis, leading to unihemispheric brain atrophy [26] during intraoperative neurosurgical monitoring in patients [27], and in acquired lesions, such as during a cerebral stroke [28,29] or following hemispherectomy [28]. MEPs can also be recorded in the ipsilateral muscles of the upper extremities in healthy subjects [30]. Ipsilateral MEPs are thought to reflect the functional activity of the uncrossed lateral corticospinal tract from the ipsilateral hemisphere [31], may reflect the activation of the cortical–subcortical–spinal pathways [32], or may be due to the activation of the crossed corticospinal tract from the hemisphere contralateral to the target limb; this is due to the proximity of the M1 cortices for lower extremity muscle representation. The functional role of ipsilateral M1 areas in MS has been associated with an adaptive response to chronic CNS injury [33,34,35]. Overall, the TMS investigation of ipsilateral MEPs in MS has not been widely considered, due to the neurophysiological mechanisms still being unknown.

Concerning the clinical use and interpretation of MEPs in diagnosing and monitoring pwMS, TMS guidelines were established by Fernández et al. in 2013 [36], referring to the TMS. This mainly included the magnetic stimulator connected to a standard EMG unit, and was less connected to linenavigated TMS implementations.

Therefore, this paper aims to review the current literature state of MEP assessment in MS. The article is organized as follows: The Section 2 examines the MEP assessment and outcome measures in MS research, assessed by TMS without navigation, TMS with line navigation, and e-field navigation TMS techniques. The Section 3 presents a two case report on MS research in MEP assessment using an e-field-navigated TMS; this is performed by also testing the correspondence of e-field-navigated TMS testing with MRI and EDSS classifications. The Section 4 presents practical and technical guiding principles for improvements to TMS studies in MEP assessment in MS.

## 2. Assessment of MEPs in Multiple Sclerosis

### 2.1. Targeting M1 with TMS without Navigation, Line-Navigated TMS and e-Field-Navigated TMS

**Figure 1:**
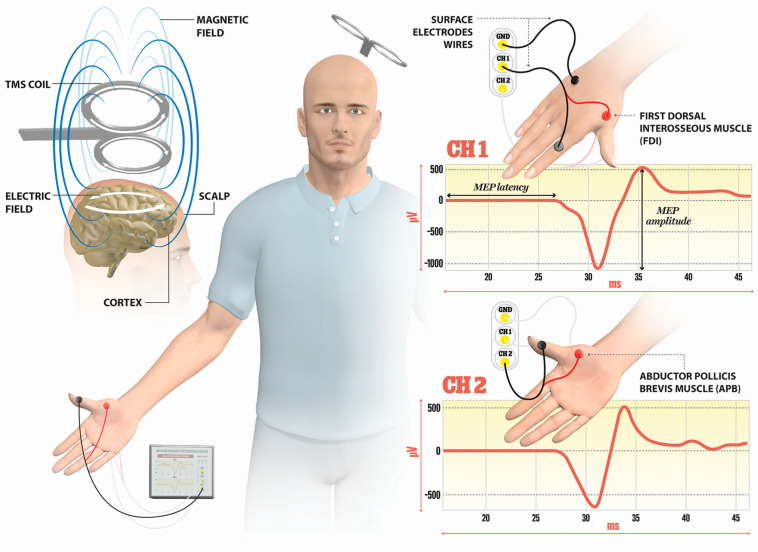
Illustration of the direction of current flows in a magnetic coil and the induced current in the brain tissue. An electric field is induced perpendicularly to the magnetic field. The magnetic coil is positioned over the M1 cortex, and surface electrodes are on the target muscles, here shown for the first dorsal interosseus (FDI) and abductor pollicis brevis (APB). Elicited MEP responses are detected at channels Ch1 for FDI, and Ch2 for APB muscle. The illustration is the property of the School of the Medicine University of Split, Department of Neuroscience, Laboratory for Human and Experimental Neurophysiology).

TMS is a noninvasive technique mainly used for the evaluation of corticospinal tract integrity and the excitability of M1 cortices in MS. The basic principle of TMS can be explained by electromagnetic induction, generating a suprathreshold current in the brain. TMS devices consist of a few circular turns of copper wire, connected to the terminals of a large electrical capacitance via a switch. A large current (monophasic or biphasic pulse configuration) of several thousand Amps flows briefly through the wire coil for less than one millisecond. The current pulse produces a rapidly changing and brief magnetic field, with a field strength similar to the static field in an MRI scanner (1–2 T). Magnetic fields generate current in the brain tissue, according to Faraday’s law of electromagnetic induction (Figure 1).

The TMS includes the magnetic stimulator connected to a standard EMG unit, with the positioning of the coil performed by using the external landmarks on the head. The determination of M1 representation for upper muscle is performed by the coil positioning; the coil is placed 5 cm lateral to the vertex along the auricular line and positioned by turning the coil approximately 45° to the parasagittal plane. In mapping the M1 representation for leg muscles, the coil is recommended to be placed over the vertex. Cervical stimulation is agreed upon by placing the coil above the C7 spinous process at the midline, or 2 cm lateral to the midline, while for the stimulation of lumbosacral roots, the coil is placed along the midline over the target vertebral body.

Line-navigated TMS is performed by placing a magnetic coil over the target area on the basis of the individual MRI image, with the maximal activation supposed to be located on the line that passes through the center of the coil perpendicular to the surface of the bottom of the coil; this is without the visualization of the spot of maximal stimulation if there is slight coil tilt [37]. Line-navigated TMS is susceptible to errors when the coil is not held continuously tangentially against the head.

E-field-navigated TMS computes the e-field maximum, where the cortex is best stimulated, online; it considers the geometry of the head, the magnetic coil shape, location, orientation, individual head shape, size, and the orientation of the cortical folds [37]. Navigated TMS combines TMS with 3D brain imaging, approximated with the spherical models, and comprises a magnetic stimulator, stereotactic camera, and integrated EMG system, including tracking tools (head tracker, coil tracker, digitizing pen). Prior to mapping M1 with navigated TMS, an MRI of the head for the subject is performed, including the MRI of the head and visible ears. After the co-registration of the subject, the reference anatomical spot for M1 for upper extremity representation is determined by the ‘‘omega knob’’ on axial MRI images, or a ‘‘hook structure’’ at the sagittal MRI [38]. The central sulcus is used as a landmark, while moving the coil in the anterior–posterior direction, to map the hot spot for M1 for the upper extremity muscle (i.e., abductor pollicis brevis, APB). When mapping the M1 for lower extremity muscles, the central sulcus is again followed as a landmark, with the posterior-to-anterior direction of the coil positioned medially over the vertex of the target hemisphere.

Line-navigated TMS and e-field-navigated TMS methods were compared in studies investigating MEPs; this was performed by stimulating the M1 area in tumor patients in preoperative settings [39], resulting in only a partial overlap in MEP maps while mapping M1 representation of upper and lower extremity muscles. The distances between the M1 motor hotspots between the two methods were 8.6 ± 4.5 mm on the contralesional hemisphere. Further, motor positive spots eliciting MEPs were significantly higher for e-field-navigated TMS, compared to line-navigated TMS. The lower rate of the positive motor hot spots detected with line-navigated TMS is probably due to a nonoptimal coil orientation and tilting with the decreased electric field at the cortex. In addition, the manual placing of the coil is more time-consuming in line-navigated TMS. Likewise, an e-field-navigated TMS can calculate and visualize the electric field online during the mapping procedure with its orientation and dose, allowing the continuous optimization of the coil positioning [39]. The final conclusions regarding the accuracy of the e-field-navigated TMS and line-navigated TMS methods are to be tested against the intraoperative golden standard direct electrical stimulation (DES) technique. Currently, e-field-navigated TMS systems have been evaluated in patients with tumors undergoing preoperative mapping of the M1 area and intraoperative DES procedures, showing a correlation between e-field-navigated TMS and DES [37].

Lastly, it is important to emphasize the variability in corticospinal excitability by mapping the M1 due to physical (tilt, location, intensity, and orientation of the coil) and physiological factors, in addition, interindividual anatomical differences in M1 that can be controlled by e-field-navigated TMS, including online calculation and visualization of an electric field [40,41], are mapped. The spatial accuracy of e-field-navigated TMS is approximately 2 mm [41], with location changes larger than 2 mm resulting in a variability of corticospinal excitability (i.e., changes in peak-to-peak MEP amplitude values), pointing to the fact that mapping of the integrity of the corticospinal tract is susceptible to small changes in physical parameters.

### 2.2. Neurophysiological Changes in the Central and Peripheral System in pwMS Investigated with TMS

The single-pulse TMS is applied for mapping the M1 and the integrity of the corticospinal tract by examining MEP outcome measures; this includes MEP latency (the transmission duration from the stimulating cortex to the onset of MEP in the EMG of the target muscle), MEP amplitude (peak-to-peak difference in MEP signal), the MEP input–output curve (I/O) (a sigmoid-shaped relation between the MEP amplitude at incremented TMS intensities), the central motor conduction time (CMCT) (the time it takes for the action potentials to travel from the site of cortical stimulation to the spinal neuron), the cortical silent period (CSP) (intracortical inhibition measure), or the resting motor threshold (RMT) (minimum intensity of stimulator output eliciting MEPs of 50 µV in at least ten trials in relaxing muscle) [17,42]. Further, short-interval intracortical inhibition (SICI), intracortical facilitation (ICF), and short-interval intracortical facilitation (SICF) can be explored if a paired-pulse TMS protocol is applied.

Recommendations for the clinical use of MEPs in MS are reported by Fernández et al. [36], and mainly discuss the application of TMS with no navigation for the use of MEP assessment in pwMS. The majority of reported studies (Table 1), assessing the MEP in pwMS, used TMS apparatus with no navigation. Table 1 presents an overview of the neurophysiological changes in the central and peripheral nervous system in pwMS when compared to healthy controls.

The findings for the neurophysiological assessment in MS, compared to healthy controls, include a prolongation in the MEP latency, an increase in the CMCT, and a decrease in the MEP amplitude, with still nonconclusive results related to RMT (findings point to be increased), CSP (findings point to be prolonged), and SICI (probably decreased) (Table 1) [17]. Two studies by Neva et al. [14] and Nantes et al. [43] used the TMS system with line navigation (neuronavigation software package by Rogue Research Inc., Canada), and a single group by Rogić Vidaković [44] used the e-field-navigated TMS to localize the M1 representation for upper and lower extremity muscles. So far, most of the MEP studies in MS have been conducted via TMS with no navigation, such as the study by Magstim, reporting the use of different coil types (circular, double-cone, figure-of-eight) (Table 1). Most of the studies included healthy controls (i.e., Pisa et al. [45]), or included the results of clinical samples of healthy controls in previously published studies. Recent reports tend to report the results of multimodal measures, including neurophysiological (MEP) assessment data, combined with MRI data on lesions, disease-related information, and clinical results of the neurological assessment (EDSS) [46].

**Table 1:** An overview of TMS studies examining the central and peripheral systems in pwMS.

Author (Year) (Reference Number)	TMS Device, Coil Type, M1 Target Location (TMS without Navigation, e-Field Navigation TMS, Navigate TMS)	Number of pwMS/HC	MEP Latency/INVESTIGATED Muscles	MEP Amplitude	RMT	CMCT	CSP	SICI
Yperman et al. (2022) [47]	Magstim 200^2^, round coil, TMS without navigation	963/	The study includes a dataset of 100,000 MEP signals in MS Metacarpal I/II, APB, metatarsal I, AH	The study includes a dataset of 100,000 MEP signals in MS	-	-	-	-
Rogić Vidaković et al. (2022) [44]	NBS navigation system (Nexstim Plc., Helsinki, Finland), figure-of-eight coil, navigated TMS, biphasic stimulation	single pwMS case report/	Prolonged MEP latencies in upper and lower extremity muscles APB, ADM, TA, AH	ns	ns	ns	ns	ns
Rogić Vidaković et al. (in review, unpublished) [48]	NBS navigation system (Nexstim Plc., Helsinki, Finland), figure-of-eight coil), navigated TMS, biphasic stimulation	23/clinical samples of healthy subjects	Prolonged MEP latencies in pwMS compared to clinical samples of HC (*p* < 0.001) APB, ADM, TA, AH	ns	ns	ns	ns	ns
Mamoei et al. (2021) [46]	Dantec Magnetic Primer TwinTop TMS & MagLite (Berlin, Germany), r-25 magnetic stimulator, circular coil, TMS without navigation	41/longitudinal study testing Fampridine responsiveness	ns VM, TA	Decreased MEP amplitude after 1 year (*p* < 0.035)	ns	CMCT prolonged after 1 year	ns	ns
Stampanon and Basssi et al. (2020) [15]	Magstim 200^2^ (Magstim Company Ltd., Spring Gardens, Whitland, UK), figure-of-eight coil, TMS without navigation	18/18	ns FDI	ns	RMT increased in pwMS compared to HC (*p* = 0.009)	ns	ns	SICI decreased in pwMS compared to HC (*p* = 0.007)
Pisa et al. (2021) [49]	Magstim 200 (Magstim Company Ltd., Spring Gardens, Whitland, UK), figure-of-eight coil, TMS without navigation	30/15	Prolonged MEP latencies compared to HC (*p* > 0.05) (posterior-anterior coil orientation) FDI	Decreased MEP amplitude compared to HC (*p* < 0.05) (posterior-anterior coil orientation)	RMT increased in pwMS compared to HC (*p* < 0.05) (posterior-anterior coil orientation)			
Pisa et al. (2020) [45]	Magstim 200 (Magstim Company Ltd., Spring Gardens, Whitland, UK), figure-of-eight coil, TMS without navigation	50/	Delayed or absent MEP to the upper limbs. MEPs bilaterally absent in the lower limbs 74% (PPMS) FDI, TA	ns	ns	ns	ns	ns
Mordillo-Mateos et al. (2019) [50]	Magstim 200 (Magstim Company Ltd., Spring Gardens, Whitland, UK), figure-of-eight coil, TMS without navigation, monophasic stimulation	17/16	ns FDI	Decreased MEP amplitude after abductions of FDI in pwMS compared to HC	RMT increased in pwMS compared to HC (*p* = 0.0139)	CMCT increased in pwMS (*p* = 0.009)	ns	ns
Zipser et al. (2018) [51]	Magstim 200 (Magstim Company Ltd., Spring Gardens, Whitland, UK), figure-of-eight coil, TMS without navigation, monophasic stimulation	13/16	ns APB	ns	RMT increased in pwMS compared to HC (*p <* 0.05)	ns	ns	ns
Neva et al. (2016) [14]	Magstim 200^2^ (Magstim Company Ltd., Spring Gardens, Whitland, UK), figure-of-eight coil, Brainsight^TM^ neuronavigation software package (Rogue Research Inc., Montréal, Canada), TMS with e-field navigation	26/11	MEP latency prolonged in pwMS compared to HC (*p* = 0.001) extensor carpi radialis	ns	RMT increased in pwMS compared to HC (*p* = 0.022)	ns	CSP onset prolonged in pwMS compared to HC (*p* = 0.011)	ns
Nantes et al. (2016) (2017) [43]	Magstim 200^2^ (Magstim Company Ltd., Spring Gardens, Whitland, UK), figure-of-eight coil, Brainsight^TM^ neuronavigation software package (Rogue Research Inc., Montréal, Canada), TMs with e-field navigation	43/29	MEP latency prolonged in pwMS compared to HC (*p* < 0.001) FDI	Decreased MEP amplitude during rest in pwMS compared to HC (*p* < 0.001)	No difference between pwMS and HC (*p* > 0.05)	ns	CSP increased in pwMS (*p* < 0.01)	No difference between pwMS and HC (*p* > 0.05)
Cabib et al. (2015) [52]	ns, figure-of-eight coil, TMS without navigation	20/13	MEP latency prolonged in pwMS compared to HC (*p* = 0.005) FDI	ns	ns	ns	ns	ns
Bridoux et al. (2015) [53]	-	12/12	ns extensor carpi radialis	Decreased MEP amplitude in pwMS (*p* = 0.03)	ns		ns	ns
Di Sapio et al. (2014) [54]	Magstim Rapid2 Device (Magstim Company Ltd., Spring Gardens, Whitland, UK), double- cone coil, TMS, without navigation	28/28	ns VM, flexor hallucis brevis, TA	ns	No difference between pwMS and HC	CMCT increased in pwMS (*p* < 0.001)	ns	ns
Von Mayenburg et al. (2013) [55]	Magstim 200 (Magstim Company Ltd., Spring Gardens, Whitland, UK), circular coil, TMS without navigation, biphasic stimulation	41/28	ns ADM, TA	ns	ns	CMCT increased in pwMS (*p* = 0.002)	ns	ns
Conte et al. (2009) [56]	Magstim (Magstim Company Ltd., Spring Gardens, Whitland, UK), figure-of-eight coil, TMS without navigation	30/17	MEP latency prolonged in pwMS FDI	Decreased MEP amplitude in pwMS (*p* = 0.001)	ns	CMCT increased in pwMS (*p* = 0.002)	ns	SICI decreased in pwMS
Firmin et al. (2012) [57]	Bistim 200 (Magstim Company Ltd., Spring Gardens, Whitland, UK), circular coil, TMS without navigation	16/29	ns ADM	ns	ns	No difference in CMCT between pwMS and HC	ns	ns
Steens et al. (2012) [58]	-	20/20	ns FDI	ns	No RMT difference between pwMS and HC (*p* = 0.18)	CMCT increased in pwMS (*p* = 0.02)	ns	ns
Morgante et al. (2011) [59]	Magstim 200 (Magstim Company Ltd., Spring Gardens, Whitland, UK), figure-of-eight coil, TMS without navigation, biphasic stimulation	33/12	ns FDI, APB	Decreased MEP amplitude in pwMS compared to HC (*p* = 0.001)	No RMT difference between pwMS and HC	CMCT increased in pwMS (*p* = 0.003)	ns	No SICI difference between pwMS and HC (*p* = 0.04)
Thickbroom et al. (2008) [60]	Magstim 200 (Magstim Company Ltd., Spring Gardens, Whitland, UK), double-cone coil, TMS without navigation	10/13	MEP latency prolonged in pwMS (*p* < 0.05) TA	No MEP amplitude difference between pwMS and HC (*p* < 0.05)	ns	CMCT increased in pwMS	ns	ns
Gagliardo et al. (2007) [61]	Magstim 200 (Magstim Company Ltd., Spring Gardens, Whitland, UK), figure-of-eight coil, TMS without navigation, monophasic stimulation	32/20	ns TA	Decreased MEP amplitude in pwMS compared to HC (*p <* 0.001)	RMT increased in pwMS compared to HC (*p* = 0.001)	CMCT increased in pwMS (*p* = 0.001)	ns	ns
Thickroom et al. (2006) [62]	Magstim 200 (Magstim Company Ltd., Spring Gardens, Whitland, UK), figure-of-eight coil, TMS without navigation	23/15	No MEP latency difference between pwMS and HC FDI	Decreased MEP amplitude in pwMS (*p <* 0.01)	ns		No CSP difference between pwMS and HC (*p* > 0.05)	ns
Liepert et al. (2005) [63]	Magstim (Magstim Company Ltd., Spring Gardens, Whitland, UK), figure-of-eight coil, TMS without navigation	16/6	ns superficial flexor digitorum	No MEP amplitude difference between pwMS and HC	ns	ns	ns	SICI decreased in pwMS (*p* < 0.01)
Mainero et al. (2004) [64]	Magstim (Magstim Company Ltd., Spring Gardens, Whitland, UK), figure-of-eight coil, TMS without navigation	12/12	ns FDI	ns	ns	CMCT increased in pwMS (*p* < 0.001)	ns	ns
Schubert et al. (1998) [65]	-	11/10	ns flexor hallucis brevis, TA	MEP area reduced in pwMS compared to HC	No RMT difference between pwMS and HC	CMCT increased in pwMS	ns	ns
Sheean et al. (1997) [66]	Magstim 200 (Magstim Company Ltd., Spring Gardens, Whitland, UK), circular coil, TMS without navigation	21/19	MEP latency prolonged in pwMS compared to HC (*p* < 0.05) adductor pollicis	No MEP amplitude difference between pwMS and HC	No RMT difference between pwMS and HC	CMCT increased in pwMS (*p* < 0.01)	ss	ns

Abbreviations: pwMS, people with multiple sclerosis; HC, healthy controls; MEP, motor evoked potentials; CMCT, central motor conduction time; RMT, resting motor threshold; CSP, cortical silent period; SICI, short intracortical inhibition; APB, abductor pollicis brevis; ADM, aductor digiti minimi; AH, abductor hallucis; FDI, first dorsal interosseus; TA, tibialis anterior; VS, vastus medialis; ns, not specified; -, information missing due to technical reasons; clinical samples of healthy subjects refers to samples of healthy controls from previous studies.

## 3. MEP Assessment in MS with e-Field-Navigated TMS

Noninvasive navigated TMS has features to show the results in real-time: the coil position, magnetic field orientation, and point on the cortex where the stimulus is delivered. The navigated system tracks the position of TMS coils relative to the subject’s head. MRI’s advanced segmentation of the individual’s brain and scalp provides 3D visualization of the subject’s head. Movements of the head are automatically detected and compensated by the tracking system, without the loss of accuracy. The induced electric field, produced by TMS, is projected onto the MRI and displays the focus of the stimulation. The applied stimuli can be presented as positive hot spot locations and repeated; this is particularly useful for monitoring purposes and longitudinal studies in pwMS. Thus, in e-field-navigated TMS, an image-guided stimulation of the M1 generates a highly accurate functional map, specific to individual anatomy. E-field-navigated TMS is frequently used for mapping the M1 cortices, as well as speech and language mappings in the preoperative setting (prior to neurosurgical procedures); this allows accurate patient risk stratification and counseling [67]. Navigated TMS may be well suited in MS research and clinical settings due to the following: (a) the precision of the system in visualizing individual brain M1 anatomy, (b) the possibility of investigating functional cortical maps for single muscles, (c) the possibility to stimulate and repeat the positive hot spot for the monitoring purposes of pwMS. Further, due to the fact that the functional pyramidal EDSS system score was shown to be the most frequently associated with sustained disability progression (in 31–51% of pwMS), followed by ≥1-point worsening in the cerebellar (35–41% of pwMS) and sensory (31–45% of pwMS) domains [7], the use of e-field-navigated TMS in evaluating corticospinal tract integrity may improve the accuracy of MEP assessment and monitoring of pwMS.

The use of e-field-navigated TMS in the assessment of MEPs can be tested in regard to the correspondence of MEP results with clinical, radiological MRI, and neurological EDSS findings in pwMS. Below, we present two opposite cases, one with a clear and the other with a non-clear correlation between TMS, MRI, and EDSS.

### 3.1. Case Study of MEP Assessment in MS Subject as an Example of Correspondence of e-Field-Navigated TMS, MRI, and EDSS Findings

**Figure 2:**
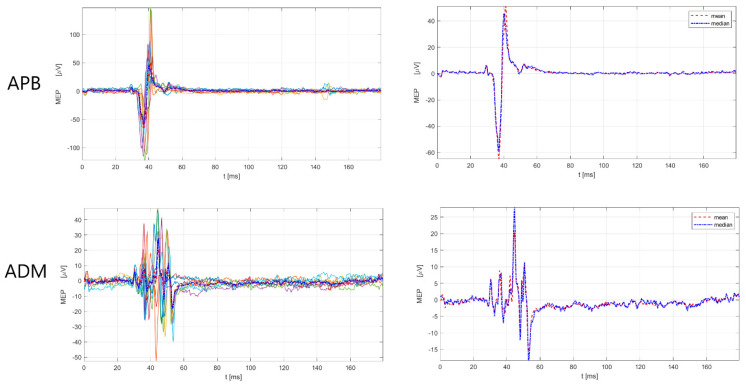
MEP responses for upper extremity (APB, ADM) muscles, right side for No3 subject. Left: superimposed responses, Right: mean and median responses. APB = abductor pollicis brevis, ADM = abductor digiti minimi. Legend: the red dashed line denotes the mean response of all MEPs, and the blue dash-dotted line represents the median response of all MEPs. MEP latency and amplitude estimation was performed by a custom-made Matlab script (R2021a) using an automatic algorithm developed by Šoda et al. (2020) [68].

**Figure 3:**
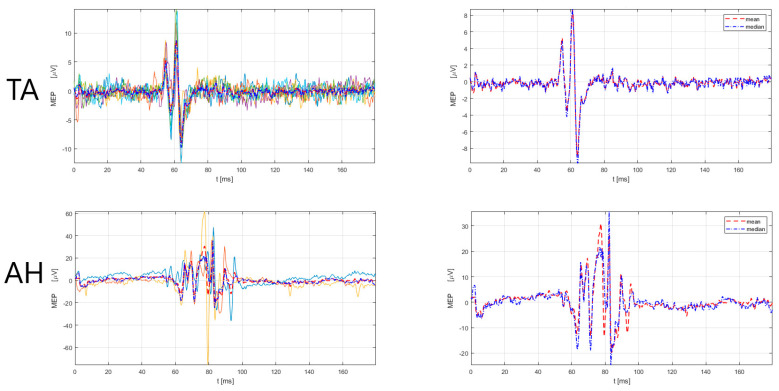
MEP responses for lower extremity (TA, AH) muscles, right side for No3 subject. Left: superimposed responses, Right: mean and median responses. TA = tibialis anterior, AH = abductor hallucis. Legend: the red dashed line denotes the mean response of all MEPs, and the blue dash-dotted line represents the median response of all MEPs. MEP latency and amplitude estimation was performed by a custom-made Matlab script (R2021a) using an automatic algorithm, developed by Šoda et al. (2020) [68].

**Figure 4:**
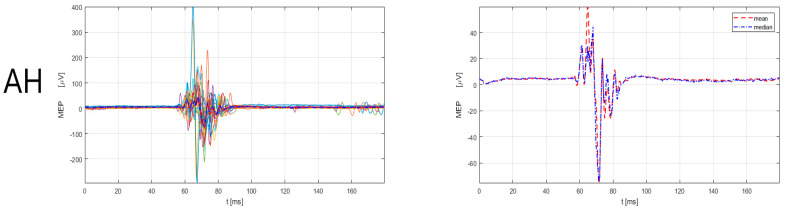
MEP responses for lower extremity (AH) muscle, left side for No3 subject. Left: superimposed responses, Right: mean and median responses. AH = abductor hallucis. Legend: the red dashed line denotes the mean response of all MEPs, and the blue dash-dotted line represents the median response of all MEPs. MEP latency and amplitude estimation was performed by a custom-made Matlab script (R2021a) using an automatic algorithm, developed by Šoda et al. (2020) [68].

The male subject (52 years old, designated as No3) had an MS duration of nine years, with a score (EDSS) of 3.5, and an EDSS functional pyramidal score of 3.0 (functional pyramidal EDSS score for the right leg of 3, a left leg of 1, right and left arm 3). Further, the patient had had six relapses since the MS diagnosis and two during the current immunomodulatory treatment with teriflunomide, with a total of six corticosteroid treatments. According to the 2017 McDonald criteria and the 2021 MAGNIMS-CMSC-NAIMS recommendations [3,4], the No3 patient had a total of 14 lesions (cortical = 0, juxtacortical = 2, periventircular = 4, infratentorial = 5). Additionally, before the e-field-navigated TMS assessment, the specific locations of the corticospinal tract were examined, including the subcortical white matter in the primary motor cortex (CST-M1), capsula interna, the cerebral peduncles and ventral parts of the midbrain and pons (CST-M2), and the ventral and lateral parts of the cervical spinal cord (CST-M3). We checked if the patient had a lesion in any of the listed locations (CST-M1, CST-M2, CST-M3), checked the number of lesions, and whether the lesion was located on the left or right side. The finding comprised four lesions in the corticospinal tract (two lesions in CST-M3 right and two lesions in CST-M3 left side). Regarding treatment, the patient had received teriflunomide for five years, and before teriflunomide, had received interferon (beta-1b) for two years. The scores on the Multiple Sclerosis Impact Scale (MSIS-29) were 48 (physical impact) and 13 (psychological impact), with higher scores indicating a more severe disease burden in the physical aspect. The electroneurographic (ENG) assessment was normal for the lower and upper extremities, including the following measures for motor nerves (n.medianus, n. ulnaris, n. peroneus, n. tibialis): distal motor latency, CMAP amplitude, conduction velocity, and F-wave latency; and for sensory nerves (n. medianus, n. ulnaris, n.suralis): sensory nerve action potential amplitude, sensory nerve action potential latency, and conduction velocity. For mapping, the M1 representation of the upper and lower extremity muscles e-field-navigated TMS system (eXima, Nexstim Plc.) was used according to the methodological procedure for M1 mapping with e-field-navigated TMS, described in paragraph 2. Table 2 presents the results of the amplitude and latency of MEPs for upper extremity muscles (APB, ADM) and lower extremity muscles (tibials anterior—TA, abductor hallucis—AH), with included referent values. The findings (Table 2) show clear prolongations of MEP latency in lower and upper extremity muscles, bilaterally, in the No3 patient. Figure 2, Figure 3 and Figure 4 present the prolonged latency of MEP responses (superimposed and mean/median) for the right, upper right, and left lower extremity muscles in this patient.

The No3 case report of MS patient provides evidence for the association between clinical EDSS and MRI findings, using the findings of the e-field-navigated TMS assessment of MEPs. Recently, the correspondence of e-field-navigated TMS and MRI findings, with EDSS scoring results, were reported by Rogić Vidaković et al. [48] (in review, unpublished) in currently unpublished work. The TMS findings classified pwMS as the same as EDSS in 70–83% of cases, and were similar or more successful than MRI, which corresponded to EDSS in 57–65% of cases (in review, unpublished).

### 3.2. Case Study of MEP Assessment in MS Subject as an Example of Non-Clear Correspondence of TMS, MRI, and EDSS Findings

**Figure 5:**
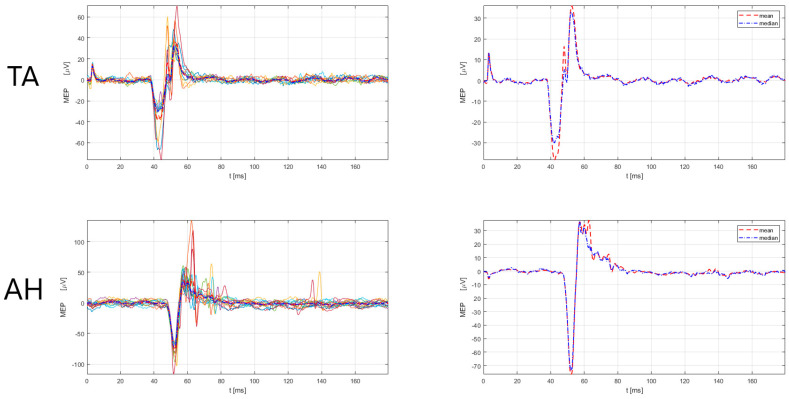
MEP responses for lower extremity (TA, AH) muscles, left side for No5 subject. Left: superimposed responses, Right: mean and median responses. TA = tibialis anterior, AH = abductor hallucis. Legend: the red dashed line denotes the mean response of all MEPs, and the blue dash-dotted line represents the median response of all MEPs. MEP latency and amplitude estimation was performed by a custom-made Matlab script (R2021a) using an automatic algorithm, developed by Šoda et al. (2020) [68].

**Figure 6:**
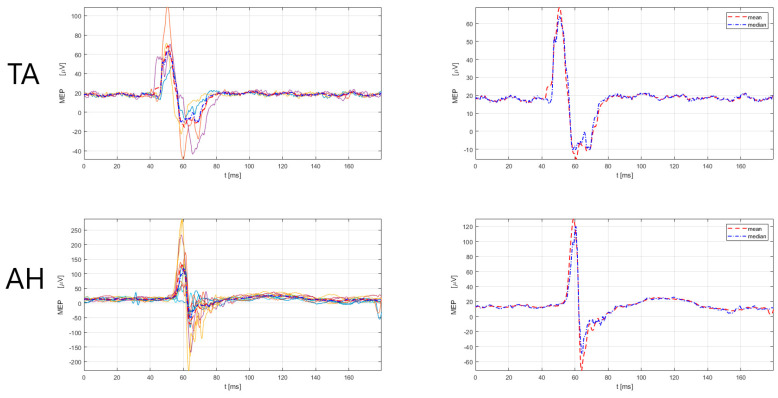
MEP responses for lower extremity (TA, AH) muscles, right side for No5 subject. Left: superimposed responses, Right: mean and median responses. TA = tibialis anterior, AH = abductor hallucis. Legend: the red dashed line denotes the mean response of all MEPs, and the blue dash-dotted line represents the median response of all MEPs. MEP latency and amplitude estimation was performed by a custom-made Matlab script (R2021a) using an automatic algorithm, developed by Šoda et al. (2020) [68].

The male subject (39 years old, designated as No5) with an MS duration of 21 years, had an EDSS functional pyramidal score of 0 (functional pyramidal EDSS score for the right leg of 0, left leg of 0, and right and left arm of 0). The patient had had five relapses since the MS diagnosis and none during the current immunomodulatory treatment with teriflunomide, with a total of four corticosteroid treatments. According to the 2017 McDonald criteria and the 2021 MAGNIMS-CMSC-NAIMS recommendations [3,4], the No5 patient had a total of 3 lesions (cortical = 0, juxtacortical = 2, periventircular = 6, spinal cord = 3). The 3 spinal cord lesions were located in the left cervical segment. The MRI findings corresponded to the TMS findings of prolonged MEP latency in the left lower extremity muscles (Figure 5), but do not correspond to the EDSS findings, which had a score of 0. Further, patient No5 had prolonged MEP latency also in the right lower extremity muscle (Figure 6), but the TMS finding did not correspond to the MRI (no lesion detected) and EDSS findings (score of 0). The findings (Table 3) show prolongations of MEP latency in lower extremity muscles, bilaterally, in the No5 patient. The slight prolongation in MEP latency of the upper extremity muscles is also detected in the upper extremity muscles, with TMS correspondence with MRI for the left upper extremity muscles and no correspondence with EDSS. TMS data on MEP also did not correspond to MRI and EDSS classifications for the right upper extremity muscles.

## 4. Discussion on Some Practical and Technical Guidelines for Improvements of TMS Studies in MEP Assessment in Multiple Sclerosis

According to recommendations from Fernández et al. [36], MEP studies are considered the first choice in patients with symptoms that are compatible with the first episode of demyelinating diseases, in patients with a clinical diagnosis of MS, and normal or inconclusive results from a brain MRI. Further, since the functional pyramidal EDSS score was shown to be the most frequently associated with sustained disability progression, it might be relevant to report functional pyramidal EDSS scores together with the overall EDSS score. In addition, with the 2017 McDonald criteria and the 2021 MAGNIMS-CMSC-NAIMS recommendations [3,4] on MRI lesion reporting, the additional inspection of corticospinal tract lesions would be suggested. It would also be recommended for TMS studies, assessing MEP in MS, to consistently report technical specifications such as EMG sampling frequency per channel, peak-to-peak amplitude, TMS pulse type, pulse width, stimulation intensity (presented as intensity value of maximal stimulator output and/or expressed in percentage in relation to RMT), and magnetic coil type. Studies using line-navigated versus e-field-navigated systems are welcomed in future studies of corticospinal tract integrity in MS, to test the accuracy of both techniques in MS research.

In research and clinical medical practice, MEP latency is a relevant neurophysiological parameter to determine the conduction time for neural impulses from the cortex to peripheral muscles. The manual latency assessment requires extensive resources and time. Thus, the importance of automatic latency estimation is emphasized. The automated latency estimation algorithms could be divided into the following categories: algorithms based on the absolute hard threshold estimator (AHTE), which are based on using hard threshold and so-called magic number [69], and algorithms that are based on statistical measures (SM), which use a standard deviation metric to find a magic number to determine the onset of the MEP [70,71,72]. Further, there is an algorithm based on the squared hard thresholded estimator (SHTE), where the coefficients of the MEP are squared and then thresholded to obtain a magic number and, thus, obtain the onset of the MEP [68]. Finally, there is an algorithm based on approximating the first derivative to find the time point of the initial deflection to determine the onset of the MEP [73]. According to the current research state, all algorithms show an accurate estimation of the latency for the peak-to-peak amplitudes greater than 100 µV. However, for the peak-to-peak amplitudes between 50 and 100 µV, the SHTE algorithm shows a slightly better accurate latency estimation than AHTE and SM, which is validated by performing a robustness test and calculating the percentage of the deviation index (PDI) [68]. We believe that there is room for improvement in latency estimation for the MEPs with peak-to-peak amplitudes of less than 100 µV; this is generally for research and clinical purposes, in order to evaluate corticospinal tract integrity. The development of such automatic algorithms would also be useful in MS due to the fact that low peak-to-peak amplitude MEPs, of less than 100 µV or less than 50 µV, could be recorded in these patients when mapping the lower extremity muscles. Still, many clinicians and researchers choose to manually determine the MEP latency and even the peak-to-peak MEP amplitude, which may lead to errors, especially if there is a larger sample of MEPs in trials.

Further, another parameter that can play the role of the biomarker of corticospinal tract integrity [74,75,76,77], and is closely related to peak-to-peak amplitudes, is the MEP I/O recruitment curve [78]. The MEP recruitment curve, or I/O curve, is a peak-to-peak amplitude versus. signal strength function. It represents the average increase in MEP amplitude (from a region of nondetectable MEPs at low stimulation strength) to an upper saturation level that, with an increase in the stimulus, can no longer increase the response [79,80]. Studies of corticospinal excitability in MS generally have not utilized the MEP I/O curves. A single study by Neva et al. [14] assessed the MEP I/O recruitment curve in 22 subjects with MS, utilizing line-navigated TMS (Magstim 2002 stimulator, Magstim Co., by means of Brainsight™ neuronavigation software package from Rogue Research Inc., Montreal, QC, Canada) and reported the linear slope of the MEP amplitude I/O curve correlation with EDSS. It is suggested that future studies could assess the MEP I/O recruitment curve in MS to convey a more comprehensive evaluation of the overall corticospinal excitability, in comparison to motor thresholds, MEP amplitude, or latency. Further, it is well known that for any given TMS strength, the generated MEPs could vary due to known [81,82,83] and still unknown reasons [84]. We believe that e-field-navigated TMS provides better options in the localization of the target region, which is important for monitoring the clinical status of the pwMS, especially in the context of mapping the M1 and functional integrity of the corticospinal pathway. In addition, MEP variability, especially related to peak-to-peak amplitude, could be reduced by using e-field-navigated TMS in MS research and clinical settings.

Finally, combining e-field-navigated TMS with electroencephalography (EEG) would enable the measurement of the brain-wide cortical reactivity to TMS in MS. The quantification of TMS-induced changes in oscillatory power, and the phase of EEG with event-related spectral perturbation and inter-trial coherence, could be investigated as an example of a measure of the cortical excitability threshold in M1 [74]. TMS–EEG is feasible for testing excitability and connectivity in cortical neural networks in MS patients, complementary to conventional EPs [50]. Still, we believe that, from the clinical point of view, combining an e-field-navigated TMS and EEG would be rather time-consuming at this point.

In addition, there are potential limits to e-field-navigated TMS, such as the accuracy and precision of the navigated transcranial magnetic stimulation [85]. When performing stimulation, possible navigation errors can occur due to distortions in image MRIs, head-to-MRI registration [86,87], localization and movement of the head tracker [88], and localization of the coil tracker [41]. Nieminen et al. [85] concluded that the head-image coordinates’ coil localization accuracy and precision, and their effect on the e-field estimation, depends on the navigation method. For example, they have found that the average coregistration accuracies were in the range of 2.2–3.6 mm and 1°, and the precision values were approximately half of the accuracy values. They recommended utilizing the surface-based approach for head-to-MRI registration and realistic e-field model computations to ensure a better navigation.

## 5. Conclusions

The functional integrity of the corticospinal pathway in MS can be investigated in two directions: medical and technical. The reason for recommending the use of an e-field-navigated TMS is to reduce technical errors [85]. By reducing technical errors, medical research that is as relevant as possible is achieved; this can ultimately result in new insights into the neurophysiological mechanisms in MS. The functional systems in MS are clinically evaluated by EDSS, with the functional pyramidal component highly correlating with sustained disability progression [7]. In addition to TMS becoming an important tool for detecting the degree of disability, by assessing the markers of corticospinal excitability in MS [14,15,43,44,45,46,47,48,49,50,51,52,53,54,55,56,57,58,59,60,61,62,63,64,65], the introduction of e-field-navigated TMS reduces errors and reproducibility, and enables a more objective testing of the correspondence with clinical EDSS and MRI data. A neurological EDSS assessment of the functional pyramidal system could be functionally verified or tested via neurophysiological e-field-navigated TMS assessment; this is because it can improve the assessment’s accuracy, leading to more the objective correspondence testing of corticospinal tract integrity by TMS and EDSS. Ultimately, the technical advantages of e-field-navigated TMS should be considered in MS research and clinical settings to improve the reliability of MEPs, especially in monitoring disease progression.

## Figures and Tables

**Table 2:** Stimulation intensity, latency, and amplitude of MEP responses recorded from upper and lower limb muscles in No3.

	Resting Motor Threshold (RMT) (Referent Values) * (%)	RMT Left Hemisphere (Right Side Extremities) (%)	RMT Right Hemisphere (Left Side Extremities) (%)	MEP Latency (ms) (Referent Values) *	Extremities Right Side		Extremities Left Side	
Muscles					MEP Latency (ms)	MEP Amplitude (µV)	MEP Latency (ms)	MEP Amplitude (µV)
APB	≤/41	36	37	21 ± 0.7	31.75	160.65	30	99.73
ADM	≤56	42	37	20 ± 0.7	30.79	69.97	30	104.50
TA	60–80	79	80	28 ± 1.2	47.08	17.87	-	-
AH	55–75	79	80	40 ± 1.5	60	82.07	58.16	284.57

Legend: abductor pollicis brevis—APB; abductor digiti minimi-ADM; tibialis anterior—TA; abductor hallucis-AH); “-” no MEP was elicited/absent MEP; % maximal stimulator intensity percentage measure Note: The table reports the mean value of 10–20 trials per extremity. The MEPs with an amplitude of lower than 50 µV (the lowest peak-to-peak amplitude of 17.87 µV) were also included because the patient could not tolerate a further increase in the stimulation intensity. Reference values were taken and adapted according to * Rossini et al. from 2015 [17]. MEP latency and amplitude estimation was performed by a custom-made Matlab script (R2021a) using an automatic algorithm, developed by Šoda et al. in 2020 [68], which can automatically detect peak-to-peak MEP amplitudes lower than 50 µV.

**Table 3:** Stimulation intensity, latency, and amplitude of MEP responses recorded from upper and lower limb muscles in No5.

	Resting Motor Threshold (RMT) (Referent Values) * (%)	RMT left Hemisphere (Right Side Extremities) (%)	RMT Right Hemisphere (Left Side Extremities) (%)	MEP Latency (ms) (Referent Values) *	Extremities Right Side		Extremities Left Side	
Muscles					MEP Latency (ms)	MEP Amplitude (µV)	MEP Latency (ms)	MEP Amplitude (µV)
APB	≤/41	51	44	21 ± 0.7	26.63	288.64	27	245.81
ADM	≤56	51	44	20 ± 0.7	25.83	112.08	25	93.87
TA	60–80	87	82	28 ± 1.2	40.00	105.31	36.40	85.90
AH	55–75	87	82	40 ± 1.5	51.98	242.43	46.45	142.63

Legend: abductor pollicis brevis–APB; abductor digiti minimi–ADM; tibialis anterior–TA; abductor hallucis-AH); % maximal stimulator intensity percentage measure. Note: the table reports the mean value of 10–20 trials per extremity. Reference values taken and adapted according to * Rossini et al. from 2015 [17]. MEP latency and amplitude estimation was performed by a custom-made Matlab script (R2021a) using an automatic algorithm developed by Šoda et al. in 2020 [68].

## Data Availability

Not applicable.
